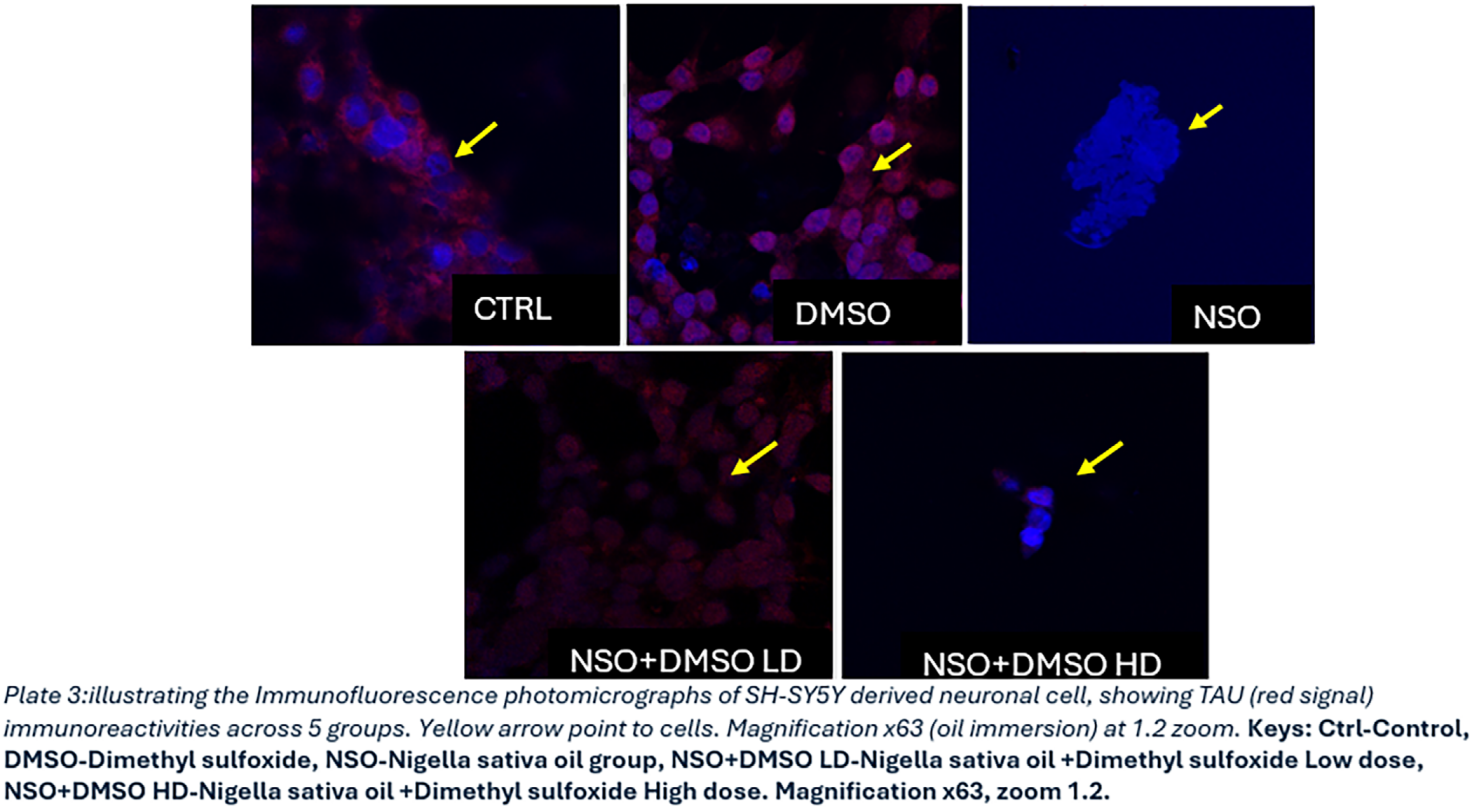# Effects of *Nigella Sativa* oil on Tau Fluorescence in Cys‐variant dGAE and SH‐SY5Y‐derived neuronal cells

**DOI:** 10.1002/alz.092476

**Published:** 2025-01-09

**Authors:** Royhaan Folarin, Moyosoreoluwa Imam

**Affiliations:** ^1^ Olabisi Onabanjo University, Sagamu, Ogun Nigeria

## Abstract

**Background:**

Alzheimer’s disease is a neurodegenerative disease associated with the accumulation of amyloid beta proteins to form plaques and the aggregation of hyperphosphorylated tau to form neurofibrillary tangles. Human fibroblast (SH‐SY5Y) cells endogenously express Tau, and the expression is further amplified upon differentiation into neuronal cells, making it a cell model of Alzheimer’s disease. *Nigella sativa* oil (NSO) contains 50% thymoquinone and has been used in the treatment of various nervous system disorders. This study therefore aimed at investigating possible inhibitory effects of *Nigella sativa* oil on the endogenously expressed tau in the neuronal SH‐SY5Y cell model of Alzheimer’s disease and on the aggregation of Tau in the Cys variant dGAE protein.

**Method:**

Amyloid fibrilization shall also be induced in vitro in NSO‐treated dGAE by 3 days of thermoshaking at 720 rpm at 37°C. Pre‐ and post‐aggregation samples were then analyzed for amyloid fibril assembly using the Thioflavin S dye and compared for fluorescence using a microplate reader. Neuronal cells were differentiated from human SH‐SY5Y cells using retinoic acid and thereafter incubated with varying concentrations of NSO. Changes in tau phosphorylation was assessed by immunofluorescence using a T‐tau antibody viewed under a Zeiss LSM780 confocal microscope.

**Result:**

While the untreated post‐aggregation dGAE samples showed higher fibrillar fluorescence than the NSO‐treated post‐aggregation samples, Tau immunofluorescence was also significantly reduced in the NSO‐treated neuronal cells.

**Conclusion:**

The Tau‐limiting effect of NSO was observed in dGAE fibrilization and in Tau immunofluorescence in SH‐SY5Y‐derived neuronal cells